# On-Chip Group-IV Heisenberg-Limited Sagnac Interferometric Gyroscope at Room Temperature

**DOI:** 10.3390/s20123476

**Published:** 2020-06-19

**Authors:** Francesco De Leonardis, Richard Soref, Martino De Carlo, Vittorio M. N. Passaro

**Affiliations:** 1 Dipartimento di Ingegneria Elettrica e dell’Informazione, Politecnico di Bari, Via Edoardo Orabona n. 4, 70125 Bari, Italy; francesco.deleonardis@poliba.it (F.D.L.); martino.decarlo@poliba.it (M.D.C.); 2 Department of Engineering, University of Massachusetts Boston, Boston, MA 02125, USA; richard.soref@umb.edu

**Keywords:** group IV photonics, quantum photonics, integrated interferometer, quantum interference, integrated quantum gyroscope, Sagnac effect

## Abstract

A room-temperature strip-guided “manufacturable” Silicon-on-Insulator (SOI)/GeSn integrated-photonics quantum-gyroscope chip operating at 1550 nm is proposed and analysed. We demonstrate how the entangled photons generated in Si Spontaneous Four Wave Mixing (SFWM) can be used to improve the resolution of a Sagnac interferometric gyroscope. We propose different integrated architectures based on degenerate and non-degenerate SFWM. The chip comprises several beam splitters, two SFWM entangled photon sources, a pump filter, integrated Mach–Zehnder interferometric gyro, and an array of waveguide coupled GeSn/Ge/Si single-photon avalanche detectors. The laser pumped SWFM sources generate the signal-idler pairs, which, in turn, are used to measure the two-photon, four-photon, and higher order coincidences, resulting in an increasing of the gyro resolution by a factor of two and four, with respect to the classical approach.

## 1. Introduction

This theoretical paper proposes and analyses a fully integrated room-temperature entangled-photon gyroscope chip that uses path-entangled N00N-states and squeezed vacuum light to provide super-resolution and phase sensitivity beyond the classical shot-noise limit. The group-IV-on-SOI (Silicon on Insulator) platform is proposed.

Silicon photonics (SiPh) has already proven to be important for actualizing several integrated quantum-photonic applications. Group IV photonics is an “expanded version” of SiPh with added capabilities in on-chip SiGeSn photodetectors and light sources. The group IV approach offers the possibility of replacing superconducting nanowire detectors with 300 K waveguide-integrated single-photon avalanche diodes (SPADs) [1]. In the present paper, the strong 1550-nm absorption capability of GeSn is employed in order to create an efficient separate absorber within such SPADs.

The novelty of the present work consists in the following aspects: (i) manufacturable SOI chip, including the monolithic integration of all parts of the quantum gyro chip; (ii) room temperature entangled-photon source using silicon Kerr-effect strip waveguides; (iii) room temperature GeSn/Ge/Si SPAD arrays that are waveguide-coupled to the interferometer section; and, (iv) an increase of 2× or 4× over classical gyro resolution using SPAD arrays and multiply-split Sagnac interferometers. At the present time, the GeSn SPADs are purely theoretical and experiments are required to verify the predicted efficient 300 K waveguide-coupled operation. Work is currently going on in several laboratories to provide this verification.

## 2. Quantum Photonics Perspective

An examination of recent literature shows that chip-scale integrated photonics provides practical realizations of complicated quantum functions, such as processing units in photonic quantum computation/simulation [2,3,4,5,6], sender/receiver modules in quantum communication [7,8,9,10,11], quantum key distribution [12,13], quantum money [14], and quantum anonymity [15], as well as quantum communication complexity [16]. The quantum photonic circuit has advantages for scalability and stability [17,18,19,20].

Practical implementations require compatible building-blocks, such as quantum light sources for biphoton state generation, optical interferometers for pump light manipulation and quantum state manipulation, and single-photon detectors for quantum state measurement. CMOS-compatible technologies promise bringing quantum photonics towards higher integration levels. However, the on-chip suppression of photonic noise leads to substantially higher pump intensity when compared to that of the photon-pairs. An externalized solution to this problem using fiber or bulk optical components hinders the compactness and stability of the system. Well-designed waveguided chips solve the compactness problem.

We have decided to use third-order nonlinear optics to attain an on-chip, room-temperature telecom-compliant quantum light source. A review of the literature shows several viable approaches, such as the device that integrates on a single substrate a non-linear photon-pair generator and a passive pump rejection filter [21], using the CMOS-compatible SiPh platform. Similarly, a hybrid Si waveguide scheme to avoid the impact of noise photons induced by pump has been investigated [22]. The scheme is composed of strip waveguide and shallow-ridge waveguide structures and utilizes the difference of biphoton spectra generated by spontaneous four-wave mixing (SFWM) in these two waveguides. Also considered is the on-chip integration of identical photon sources with reconfigurable waveguide circuits. There, an SOI device that combines two four-wave mixing sources in an interferometer with a reconfigurable phase shifter has been proposed [23,24]. More recently, an array of Si micro-resonator sources has been proposed [25]. Higher-dimensional quantum states using the higher-order radial modes of a micro-disk resonator coupled with an integrated waveguide has been also investigated [26]. The quantum source approach followed in this paper is the non-resonant SFWM.

## 3. Quantum Photonics for Rotation Sensing

Photonic chips for quantum metrology and quantum sensing are also quite feasible. However, quantum photonic sensing has been less explored than the quantum photonic applications discussed above. In particular, the quantum gyroscope chip is a new frontier.

Looking at the prior art, optical gyroscopes that are based on the Sagnac effect and optical interference have proven to be an invaluable tool in sensing and navigation. The Sagnac effect refers to the relative phase $\varphi_{S}\left(\Omega \right)$ experienced by counter-propagating light waves in a rotating interferometer [27]. The effect allows us to determine the absolute rotation Ω with respect to the inertial space [28] and it has found application in navigation systems for spacecraft [29] and aircraft [30], as well as self-driving vehicles, such as autonomous cars [31]. Many of the above-mentioned applications have been realized by inducing the Sagnac effect in either a fiber-optic interferometer gyro (IFOG) or a ring resonator fiber gyro (RFOG). Although the fiber optic gyros guarantee high performances due to their capability of increasing the optical path [32], they have the drawback of not being fully integrable on a single chip, i.e., their package is not compact. Thus, it is natural to consider the integrated photonic gyro as an alternative to IFOG and RFOG, at least in some applications. The potentiality of integrated optical gyroscopes in both passive and active configuration has been outlined in a review paper [33]. Recently, non-Hermitian systems have gained interest in the field of integrated optical gyroscopes. In particular, the sensitivity enhancement of an optical gyroscope working near an exceptional point has been studied in both parity-time [34] and anti-parity-time [35] symmetric microscale optical gyroscopes. The enhanced Sagnac resonance splitting between two counterpropagating modes has been shown to be independent of the dimensions of the device, leading to the possibility of realizing integrated optical gyroscopes with increased sensitivity with respect to classical Sagnac ones. The effect has been recently experimentally demonstrated in a Brillouin-based integrated optical gyroscope [36] working near its exceptional point and in an RLG-based single-cavity parity-time symmetric gyroscope [37].

The most recent research efforts in the field of optical gyros are concerned with the reduction of noise components to improve the device resolution. High efficiency techniques of signal processing after the photo-detection step have been pointed out to compensate all of the errors by using a feedback system, especially in the IFOG based system [32]. However, even under ideal conditions, where all error sources can be compensated, the uncertainty in the measurement of $\varphi_{S}$ is limited by the shot noise, which is caused by the quantization of the electromagnetic field itself. Therefore, this uncertainty $\delta \varphi_{S}$ is given by the ratio between the photon shot noise and the interference fringe slope and this is minimum where the fringe slope is maximum. In other words, $\delta \varphi_{S}=\pi /\left(\sqrt{n_{ph}\eta_{D}\tau }\right)$ where $n_{ph}$ is the number of photons/sec falling on the detector, $\eta_{D}$ is the quantum efficiency of the detector, and τ is the averaging time, depending on the bandwidth of the detection system [32]. Therefore, the shot-noise limit (SNL) constitutes the fundamental boundary for the phase resolution ($\Delta \varphi_{S}$) achievable with coherent or thermal states. In this context, the phase resolution can obviously be enhanced by increasing the average photon rate at the detector. However, large values of optical power could induce additional phase noise that results from detrimental effects, like the non-linear Kerr effect or coherent back-scattering [32,33]. As a result, a trade-off between these additional noise sources and the SNL has to be made to find the optimal operating point in any practical interferometric gyro.

This is true in the case of interferometric gyros under the classical state of light. However, quantum metrology provides a way to improve the precision of measurement beyond the levels obtained in the classical domain [38]. Specifically, the sensitivity of the Sagnac interferometer could be considerably improved by using correlated photons. Indeed, the path-entangled N00N-states leads to an attainment of a shortened de-Broglie wavelength λ/N (λ is the physical wavelength of the individual photons), resulting in an increase of the interferometric fringe pattern by a factor of N (super-resolution) without changing the physical wavelength of the photons [39]. In this context, the theoretical performances of the fiber quantum gyro have been presented [40], where the entangled photons that are produced in parametric down conversion have been used to improve the resolution of a Sagnac interferometer. Those authors have demonstrated that two-photon and four-photon coincidences increases the resolution by a factor of two and four, respectively. Very recently, the interference between counter-propagating modes in the IFOG at different rotational speeds, using the canonical two-photon N00N state (N = 2), has been experimentally demonstrated. This study represents an important step towards the achievement of the super-resolution IFOG [41].

Some theoretical aspects of the present work are similar to those that are presented in the literature [40,41]. However, here we have made significant changes, improvements, and expansions, as follows: (1) we adopted an integrated approach that is arguably more amenable to low-cost high-volume manufacture, compact package, and reduced size than is the fiber optic platform using discrete optics; (2) we operate around 1550 nm using the Spontaneous Four Wave Mixing (SFWM) as a source of entangled photons instead of the Spontaneous Parametric Down Conversion (SPDC), where the pump photons at 405 nm are converted into pairs of signal and idler photons at 810 nm [40]; (3) we operate with pump, signal, and idler photons having the same state of polarization rather than horizontal and vertical polarization [40,41]; (4) we adopted the CMOS-compatible integrated quantum photonic platform operating at the 1550 nm telecom wavelength, however the proposed device is also suitable to operate if desired at the “new” 2000-nm optical wavelength [42]; (5) our device uses strip-channel waveguides that are based on Silicon material, integrated beam splitter (BS), integrated Mach Zehnder interferometer (MZI), rather than fiber-optic bulk beam splitter, mirrors, half wave plate, and polarizing beam-splitter (our components offer reduced size and weight); and, (6) monolithic integration of quantum gyro and Single Photon Avalanche Photodetectors (SPADs) is proposed, rather than the hybrid and discrete solution. In this context we adopt the waveguide-integrated SPAD solution proposed in our previous work [1], based on GeSn-on-Si platform. Indeed, when compared with present Ge-on-Si SPADs, the GeSn-on-Si SPAD gives better wavelength coverage due to its selectable bandgap. The 1550-nm GeSn-on-Si SPAD has stronger absorbance, higher SPD efficiency, and lower spatial volume. Another potential advantage of our approach is that hundreds or thousands of SPADs could be closely integrated on-chip at a low cost of production.

## 4. Numerical Results

The goal of this section is to theoretically demonstrate the super-resolution beyond the shot-noise limit for several interferometric quantum gyro architectures. In this context, we will adopt the main building blocks well standardized in the silicon quantum photonic platform. Indeed, it is largely recognized that the photonic platform represents an efficient way to realize complicated quantum functions, such as the applications described in the previous section.

### 4.1. Device Overview

**Figure 1:**
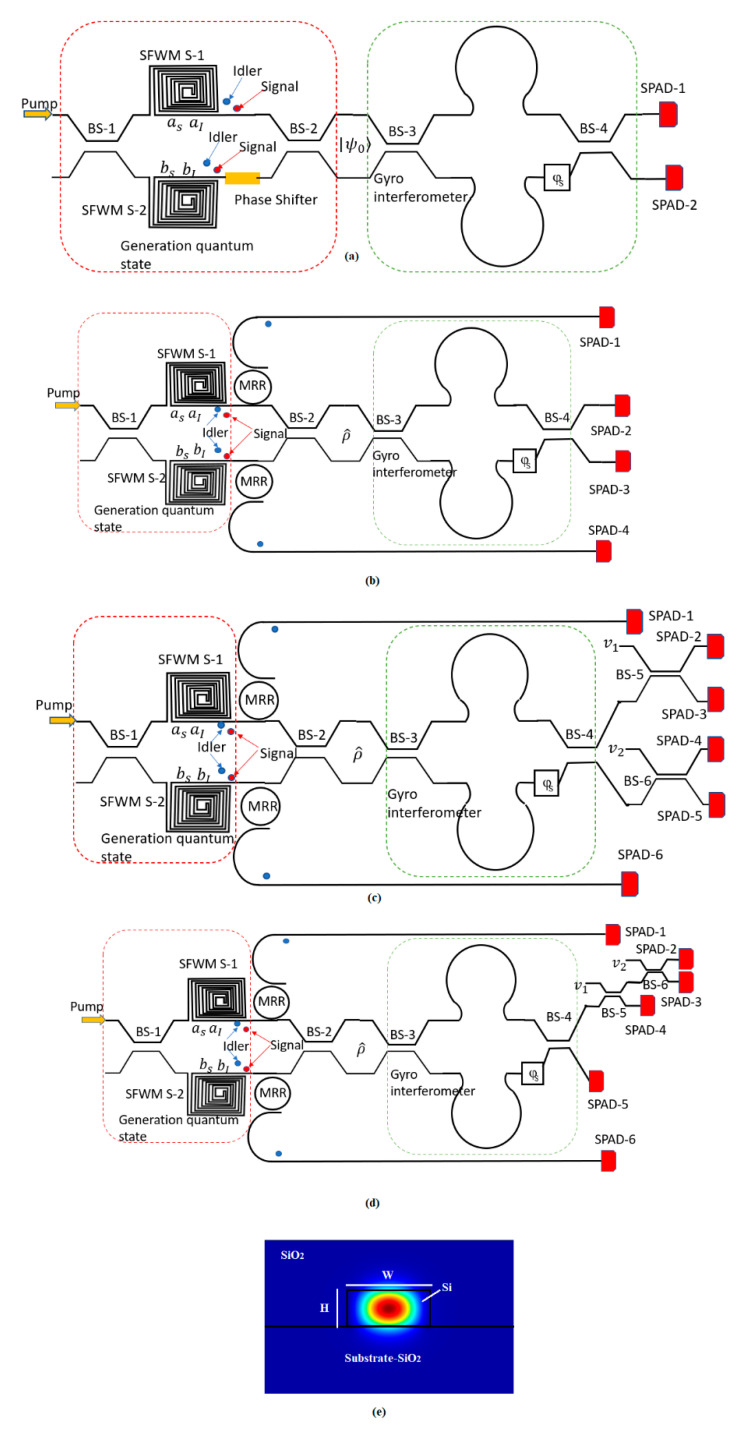
Schematic top view of proposed quantum optical gyro architectures with 2× (**a**–**b**), and 4× (**c**–**d**) advantage; and, (**e**) Poynting vector for TE mode at 1550 nm, assuming H = 220 nm and W = 500 nm.

Figure 1a–d show four proposed integrated-photonic chip architectures and operation schemes. Figure 1a,b are two alternative solutions giving the same resolution, while Figure 1c,d are designed to give higher resolution than Figure 1a,b. In each case, the chip area consists of three waveguide-connected sections for: (i) generation of quantum state, (ii) the interferometric gyro, and (iii) high-efficiency detection and analysis. Basically, in the generation section, the photonic circuit consists of a beam splitter (BS-1) to couple the pump laser to the entangled photon sources, two photon-pair sources (S-1 and S-2), each comprising a long spiraled Silicon strip waveguide in which the SFWM process is induced; S-1 and S-2 realize the two arms of a MZI that is composed of two beam splitters (BS-1, BS-2) and a phase-shifter, inducing the phase θ. The gyro transducer is realized by means of an interferometric MZI composed of long semi-circular arms (to increase the Sagnac scale factor, see Equation (1)) and by two beam splitters (BS-3, BS-4). The phase shift that is induced by the angular rotation is represented by the box with $\varphi_{S}\left(\Omega \right)$ (see Figure 1). The filtering stage for the residual pump is not shown in the architectures of Figure 1a–d, for editing reasons. However, we think that a micro-ring racetrack resonator coupled on both sides to silicon bus waveguides can be considered to be an efficient choice and can then be implicitly assumed in our devices. Typically, $\varphi_{S}\left(\Omega \right)$ is considered to be a consequence of the difference in the arrival time between the clockwise and counterclockwise propagating waves. Thus, $\varphi_{S}\left(\Omega \right)$ is given in Equation (1), where A is the interferometer area. In particular, a more general approach [32] shows that the phase shift does not depend on the shape of the interferometer and it is proportional to the flux of the rotation vector Ω through the interferometer enclosed area.

(1) $$\varphi_{S\;}=\frac{8\pi }{c\lambda }A\Omega$$

Classically, the phase shift $\varphi_{S}\left(\Omega \right)$ is evaluated by means of the interference pattern, proportional to $cos^{2}\left(\varphi_{S}/2\right)\;$.

Here, we demonstrate how the proposed quantum gyro devices can operate beyond the classical resolution. The SFWM process creates a signal-idler photon pair (s,I) by annihilating two photons from a bright pump beam. Non-degenerate pairs are created by a single wavelength pump (p) , while degenerate pairs require a dual wavelength pump scheme $\left(p_{1},p_{2}\right)\;$. In our gyro devices, we consider both of the approaches. The energy conservation requires $2\omega_{p}=\omega_{s}+\omega_{I}$ or $2\omega_{s}=\omega_{p1}+\omega_{p2}$ for the non-degenerate and degenerate cases, respectively. In the following analysis, we set $\lambda_{p\left(s\right)}=\lambda_{0\;}$, and $\omega_{p}-\omega_{s}=\omega_{I}-\omega_{p}=\Delta \omega$ $(\omega_{p2}-\omega_{s}=\omega_{s}-\omega_{p1}=\Delta \omega$) in the case of a non-degenerate (degenerate) SFWM process. It is well known that an efficient FWM process requires the phase-matching condition κ  = 0 to be met, where κ is the net phase mismatch as: $\kappa =\Delta \beta +\gamma \left(P_{1}+P_{2}\right)$ with $\gamma \left(P_{1}+P_{2}\right)$ the phase mismatch due to nonlinear Kerr effects. The terms γ, $P_{1}$ and $P_{2}$ indicate the non-linear parameters depending on the Kerr refractive index $n_{2}$ and the two pump powers, respectively. It holds $P_{1}=P_{2}=P_{0}$ for the non-degenerate case. The parameter $\Delta \beta =\beta_{s}+\beta_{I}-2\beta_{p}$ ($\Delta \beta =2\beta_{s}-\beta_{p1}-\beta_{p2}$) is the mismatch due to both material and waveguide dispersion for the non-degenerate (degenerate) case, being numerically evaluated by means of full vectorial FEM simulations, including the Sellmeier equation for Si and SiO_2_. Among different excitation methods, the most efficient way to realise the condition κ = 0, is to operate in the anomalous Group Velocity Dispersion (GVD) region.

**Figure 2:**
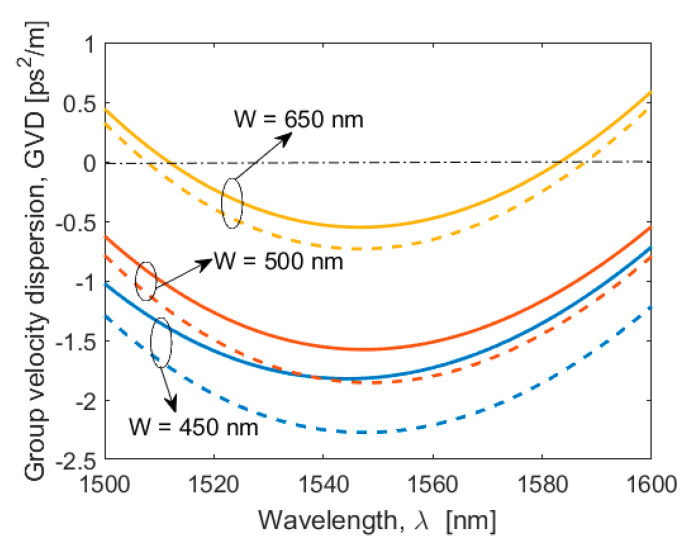
Group Velocity Dispersion (GVD) coefficient spectrum for different values of the SOI waveguide width, assuming H = 220 nm (solid line) and H = 250 nm (dashed line).

**Figure 3:**
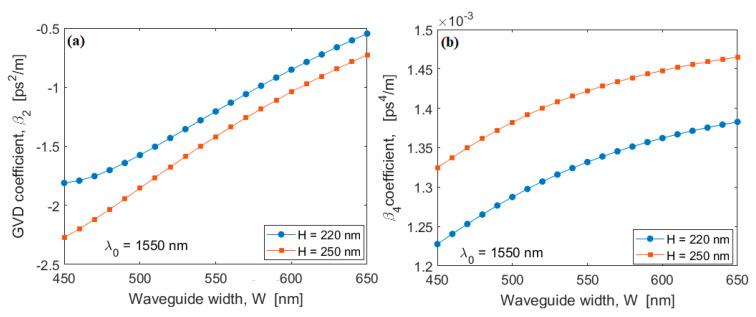
(**a**) GVD coefficient at 1550 nm as a function of the SOI waveguide width for H = 220 nm and H = 250 nm; (**b**) Fourth order dispersion coefficient at 1550 nm as a function of the waveguide width for H = 220 nm and H = 250 nm.

In general, low-loss PICs are fundamental in quantum photonic applications. In this context, low-loss waveguides have been manufactured in several platforms, such as Silicon, Silicon Nitride, and Silicon Dioxide [43]. Recently, a silicon-rich nitride core waveguide has been proposed and experimentally demonstrated for non-linear applications to fill the gap between the pure silicon waveguide and the pure silicon nitride waveguide with respect to the non-linear properties [44,45,46]. The manufacturing process of this platform is CMOS compatible and the increased silicon content allows for tensile stress reduction. Moreover, the silicon-enriched nitride presents a measured non-linear Kerr coefficient $n_{2}$ of 1.4 × 10^−18^ m^2^/W (five times higher than stoichiometric silicon nitride) and a refractive index of 2.1 at 1550 nm that enables high optical field confinement, allowing high intensity nonlinear optics and light guidance, even with small bending radii. Despite these advantages, an increase of the propagation loss from 8.4 × 10^−4^ dB/cm up to 1 dB/cm is recorded, when changing the SiN platform from stoichiometric to silicon enriched, respectively. However, here, we mainly focus on foundry-compatible and CMOS-compatible silicon waveguides. The loss coefficient of 2 dB/cm is commonly adopted for silicon strip waveguides [22] manufactured with standard CMOS process. However, a significant improvement in the propagation loss has been experimentally reported [47], where a record low coefficient of 0.45 dB/cm ± 0.12 dB/cm (adopted in the following simulations) has been obtained by means of advanced CMOS processes. In this context, we investigate the two photon-pair source performances for SOI platforms. The silicon waveguide is a strip structure having height H, width W deposited on the silicon dioxide layer. The cladding is assumed to be SiO_2_. With the aim of designing the waveguide cross section to induce the phase matching conditions between pumps, signal, and Idler waves, we evaluate the SOI GVD coefficient ($\beta_{2}$) and the fourth-order dispersion coefficient ($\beta_{4}$) as a function of wavelength, waveguide width, and two values of the height, H = 220 nm and H = 250 nm. The simulations have been performed by means of a commercial software based on full-vectorial FEM [48]. Figure 2 shows the GVD coefficient spectrum in the range 1500 nm–1600 nm, for different values of W and assuming the quasi-TE polarization state. For each case considered, the quasi-TE GVD coefficient presents an anomalous region for wavelengths around 1550 nm, recording larger values for H = 250 nm. Moreover, in the wavelength range considered, two Zero Group Velocity Dispersion (ZGVD) points ($\lambda_{z1}$ and $\lambda_{z2}$, with $\lambda_{z1}$ < $\lambda_{z2})$ are achieved for H = 220 nm and W > 575 nm, or H = 250 nm and W > 590 nm (in Figure 2 we have only presented the case for W = 650 nm). In addition, the simulations indicate that $\lambda_{z1}$ and $\lambda_{z2}$ show a red and blue shift, respectively, as the waveguide width is increased. Figure 3a,b show the $\beta_{2}$ and $\beta_{4}$ coefficients at $\lambda_{0}$ = 1550 nm as a function of the waveguide width, for H = 220 nm and H = 250 nm, respectively. The plots indicate that it is possible to realize the perfect phase matching condition, since $\beta_{2}$ is negative and $\beta_{2}$ and $\beta_{4}$ present opposite signs.

**Figure 4:**
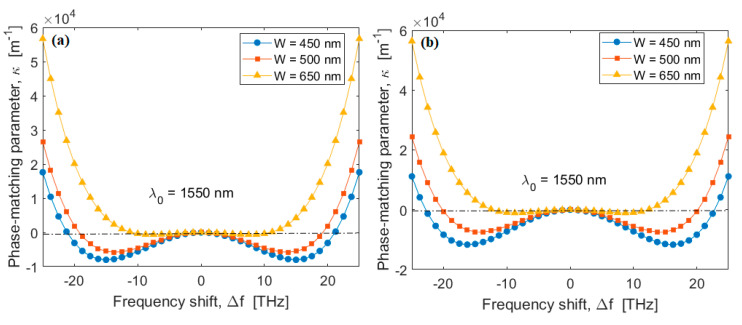
Phase matching parameter as a function of the frequency shift for different values of the SOI waveguide width. (**a**) H = 220 nm; (**b**) H = 250 nm.

In this context, Figure 4a,b show the phase matching parameter κ as a function of frequency shift Δf=Δω/2π at $\lambda_{0}$ = 1550 nm ($\lambda_{p}=\lambda_{0}$ for non-degenerate SFWM and $\lambda_{s}=\lambda_{0}$ for degenerate SFWM), for different values of W, and assuming H = 220 nm and H = 250 nm, respectively. In the simulations, a continuous wave pump having $P_{0}$ = 10 mW is assumed inside the spiral waveguides. The curves indicate that the zero crossing (κ = 0) takes place for two values of |Δf| ($\left|\Delta f_{0,1}\right|$ and $\left|\Delta f_{0,2}\right|\;$, with $\left|\Delta f_{0,1}\right|$ < $\left|\Delta f_{0,2}\right|$) increasing while the value of W decreases. It is worth noting that the phase matching condition takes also into account the effect of forth order dispersion effect ($\beta_{4}$). For larger levels of pump power, the phase mismatch due to the non-linear effect increases, which results in an increasing of $\left|\Delta f_{0,1}\right|$ and $\left|\Delta f_{0,2}\right|$ that is needed to realize the condition κ. However, to meet the experimental needs to operate with relatively narrow Δf [23,24], we set the phase matching condition at |Δf| = $\left|\Delta f_{0,1}\right|$. The plots of Figure 4a,b record values $\left|\Delta f_{0,1}\right|$ that range between 232 GHz (200 GHz) and 362 GHz (304 GHz), when changing W from 450 nm to 650 nm, for H = 220 nm (250 nm).

### 4.2. Nonlinear Source and State Preparation

**Figure 5:**
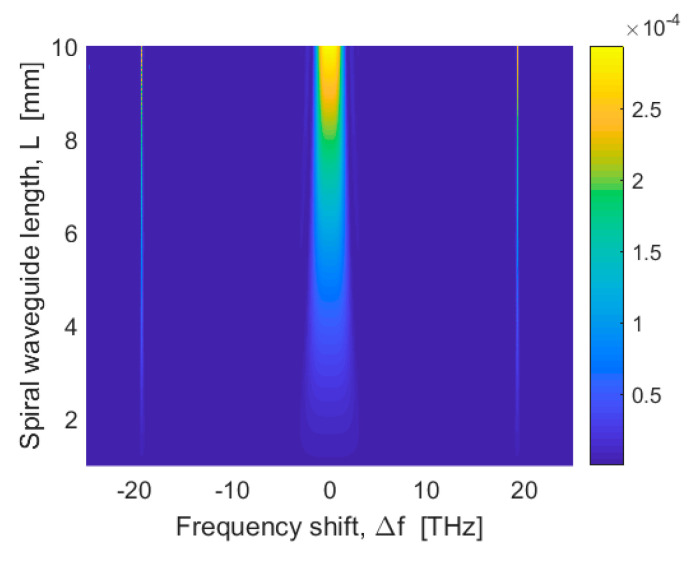
Photon pair generation rate as a function of the spiral waveguide length and frequency shift.

Hereafter, we assume the following waveguide cross sections: H = 220 nm, W = 500 nm as a trade-off choice. In this context, we assume for the non-degenerate SFWM process: $P_{0}$ = 10 mW, $\lambda_{p}$ = 1550 nm, Δf = 240 GHz, $\lambda_{s}$ = 1551.9 nm, and $\lambda_{I}$ = 1548.1 nm. Similarly, for the degenerate SFWM process, we have: $\lambda_{s}$ = 1550 nm, $\lambda_{p1}$ = 1551.9 nm, and $\lambda_{p2}$ = 1548.1 nm. Figure 5 shows the colour map of the photon pair generation rate for non-degenerate SFWM sources, as a function of the spiral waveguide length and frequency shift, as obtained through Equation (1) of ref. [49], where the modulation instability has been taken into account. However, it is worth outlining that this equation degenerates in the expression of the square of squeeze parameter (${\left|\xi \right|}^{2}$), when the condition $\gamma P_{0}L\ll 1$ is satisfied. In the simulations, we have assumed a pump power of 10 mW inside the spiral and the photon pair generation rate, depending on the effective length ($L_{eff}=\left(1-e^{-\alpha L}\right)/\alpha$, where α is the Si loss coefficient) instead of the geometrical length L. The sharp peaks that are placed at ±19.27 THz are induced by the perfect phase matching occurring for |Δf| = $\left|\Delta f_{0,2}\right|$ (see Figure 4a). In Figure 5, the full width half maximum (FWHM) of the photon pair generation rate ranges between 3.49 THz and 2.52 THz, when changing L from 5 mm to 10 mm, respectively.

Similar investigations have been performed for SiN material, where SiN strip waveguides having height *H* and width *W* deposited on the silicon dioxide layer have been considered, for both stoichiometric and Si-enriched SiN platforms. The results are summarized in Table 1, which indicates the superiority of SOI in terms of the photon generation rate. Moreover, the SOI platform does not suffer from the spontaneous Raman scattering, which is a significant problem in Silicon Nitride waveguides.

With reference to Figure 1a, the quantum state at the output of the generation section is given in the form [23]:

(2) $$|\psi_{0}\rangle =\cos\left(\theta \right)|\psi_{bunch}\rangle +\sin\left(\theta \right)|\psi_{split}\rangle$$

where θ is the phase induced by the phase shifter. In Equation (2), the $|\psi_{bunch}\rangle$ is the quantum state describing the photons bunched together in either output mode A (top waveguide) and B (bottom waveguide). Conversely, $|\psi_{split}\rangle$ represents the state for which one photon is in each mode. As detailed in [23], in the general case of non-degenerate SFWM, the bunch and split states occur with a probability $P_{bunch}$ and $P_{split}$, depending on the phase θ , as given in Equation (3):

(3) $$\{\begin{array}{c} P_{split}={\left|\left(\Gamma_{0}+\Gamma_{in}\right)\sin\left(\theta \right)\right|}^{2}\\ P_{bunch}^{A_{B}}={\left|\left(\Gamma_{0}+\Gamma_{in}+\Gamma_{out}\right)\cos\left(\theta \right)\mp \left(\Gamma_{in}+\Gamma_{out}\right)\right|}^{2} \end{array}$$

where the coefficients $\Gamma_{0}$ $\Gamma_{in}$ and $\Gamma_{out}$ are defined as in [23] and depend on the squeeze parameter that is induced by the SFWM effect inside the spiral waveguide sources, input and output waveguides (BS-1 and BS-2). The numerical simulations for $P_{bunch}$ and $P_{split}$ give the similar information of Figure 5 and are then not reported here for compactness reasons (the same for the degenerate case).

According to Figure 1a, now we analyse the quantum gyro performances, while assuming the degenerate case. After normalization, the quantum state at the input of the gyro interferometric section can be written as:

(4) $$|\psi_{0}\rangle =\cos\left(\theta \right)\frac{1}{2}\left(a^{†}a^{†}-b^{†}b^{†}\right)|00\rangle +\sin\left(\theta \right)a^{†}b^{†}|00\rangle$$

where $a^{†}$ (a), $b^{†}$ (b) are the creation (annihilation) operators for photons in spatial mode A and B, respectively. The operators at the gyro output are given by:

(5) $$\left[\begin{array}{c} c\\ d \end{array}\right]=\left[\begin{array}{cc} -j\sin\left(\frac{\varphi_{S}}{2}\right)+\cos\left(\frac{\varphi_{S}}{2}\right)\cos\left(2\theta_{BS}\right) & j\cos\left(\frac{\varphi_{S}}{2}\right)\sin\left(2\theta_{BS}\right)\\ j\cos\left(\frac{\varphi_{S}}{2}\right)\sin\left(2\theta_{BS}\right) & j\sin\left(\frac{\varphi_{S}}{2}\right)+\cos\left(\frac{\varphi_{S}}{2}\right)\cos\left(2\theta_{BS}\right) \end{array}\right]\left[\begin{array}{c} a\\ b \end{array}\right]$$

where the global phase $e^{j\varphi_{s}/2}$ has been dropped. The phase $\theta_{BS}$ determines the splitting ratio of the beam splitter BS-3 and BS-4, which is assumed to be equal (design details are shown in the Supplementary Materials).

### 4.3. Quantum Interferometer and Coincidence Measurements

In the Heisenberg picture, the expectation values of photon numbers at SPAD-1 and SPAD-2 are given as:

(6) $$I_{1}=\langle \psi_{0}|c^{†}c|\psi_{0}\rangle$$

(7) $$\;I_{2}=\langle \psi_{0}|d^{†}d|\psi_{0}\rangle$$

By developing this, we have that the single photon counts at each detector is $I_{1}$ = $I_{2}$ = 1, thus no interference pattern can be measured. In this sense, it can be useful for calculating the two photon coincidences at the SPAD-1 and SPAD-2, as:

(8) $$\begin{array}{c} I_{12}=\langle \psi_{0}|c^{†}d^{†}cd|\psi_{0}\rangle \\ =\cos\left(\theta \right)\left(\cos\left(\theta \right)\sin^{2}\left(\phi_{S}\right)-\frac{1}{2}\sin\left(\theta \right)\sin\left(2\phi_{S}\right)\right)+\sin\left(\theta \right)\left(\sin\left(\theta \right)\cos^{2}\left(\phi_{S}\right)-\frac{1}{2}\cos\left(\theta \right)\sin\left(2\phi_{S}\right)\right) \end{array}$$

Equation (8) shows that for bunch (θ=0) and split (θ=π/2) quantum states, the two photon coincidence is equal to $\sin^{2}\left(\varphi_{S}\right)$ and $\cos^{2}\left(\varphi_{S}\right)$, respectively, inducing the two-fold improvement in the fringe pattern with respect to the classic approach.

In the following, we discuss how the multi-pairs generation can increase further the gyro resolution performance. In this context, we adopt the gyro configurations, as sketched in Figure 1b–d, and operating with the non-degenerate SFWM process. The architectures include two micro-ring resonators in order to filter out the idler photons generated from the source S-1 and S-2. As a result, the idler photons, detected at the SPAD-1 and SPAD-4 (architecture of Figure 1b) or at the SPAD-1 and SPAD-6 (architecture of Figure 1c,d), are used to herald the presence of signal photons at SPAD-2 and SPAD-3 or at SPAD-2, SPAD-3, SPAD-4, and SPAD-5. It is worth outlining that our architecture of Figure 1b is similar to the configuration proposed in [24] where the authors have experimentally demonstrated the high visibility on-chip quantum interference. However, here we discuss the quantum interference under the Sagnac effect and use Figure 1b as a starting point to propose and analyse the potential of the improved architecture of Figure 1c,d. It is worth noting that although it is possible to particularize the architectures of Figure 1b–d in order to operate in the degenerate mode, avoiding the presence of micro-ring resonators, we will not analyse this approach because the single mode squeezing is less efficient with respect to the two-mode squeezing. Consequently, fewer pairs for higher pump power are provided by the degenerate SFWM process. In this context, the reduced density matrix for the heralded signal photons for each source is:

(9) $${\hat{\rho }}_{i}=N_{i}\left(1-x_{i}\right)\left(x_{i}L\left(1_{Ii}\right)|1\rangle \langle {1|}_{si}+x_{i}^{2}L\left(2_{Ii}\right)|2\rangle \langle {2|}_{si}\right)$$

with i=1,2. The term $N_{i}$ is the normalization coefficient, $x_{i}$ is the squeezing parameter, and $L\left(n_{Ii}\right)$ takes into account the losses that are experienced by n idler photons emitted by the i-th SFWM source and lumped with the SPAD loss. In this context, the density matrix for the system with S-1 and S-2 is given by:

(10) $$\begin{array}{c} \hat{\rho }={\hat{\rho }}_{1}⊗{\hat{\rho }}_{2}=N_{1}N_{2}\left(1-x_{1}\right)\left(1-x_{2}\right)\\ \left(x_{1}L\left(1_{I1}\right)|1\rangle \langle {1|}_{s1}+x_{1}^{2}L\left(2_{I1}\right)|2\rangle \langle {2|}_{s1}\right)⊗\left(x_{2}L\left(1_{I2}\right)|1\rangle \langle {1|}_{s2}+x_{2}^{2}L\left(2_{I2}\right)|2\rangle \langle {2|}_{s2}\right) \end{array}$$

Thus, with reference to Figure 1b, the probability of detecting four-fold coincidences is:

(11) $$P^{Ind\left(Dis\right)}=\overset{2}{\underset{i,j=1}{\sum }}{\langle i|}_{D2}{\langle j|}_{D3}\left({\hat{\rho }}^{Ind\left(Dis\right)}\right){|i\rangle }_{D2}{|j\rangle }_{D3}$$

where the subscripts $D_{2}$ and $D_{3}$ indicate the quantum state at the input of SPAD-2 and SPAD-3, respectively, while the superscripts Ind and Dis signify the indistinguishable and distinguishable (separate temporal mode) emitted photons.

By substituting Equation (11) into Equation (10), we obtain:

(12) $$\begin{array}{c} P_{4F}^{Ind}=A_{1}{\left(\cos^{2}\left(\frac{\varphi_{S}}{2}\right)-\sin^{2}\left(\frac{\varphi_{S}}{2}\right)\right)}^{2}\\ +A_{2}\left[{\left(\sin^{3}\left(\frac{\varphi_{S}}{2}\right)-2\sin\left(\frac{\varphi_{S}}{2}\right)\cos^{2}\left(\frac{\varphi_{S}}{2}\right)\right)}^{2}+{\left(\cos^{3}\left(\frac{\varphi_{S}}{2}\right)-2\sin^{2}\left(\frac{\varphi_{S}}{2}\right)\cos\left(\frac{\varphi_{S}}{2}\right)\right)}^{2}\right] \end{array}$$

(13) $$\;\begin{array}{c} P_{4F}^{Dis}=A_{1}\left[\cos^{4}\left(\frac{\varphi_{S}}{2}\right)+\sin^{4}\left(\frac{\varphi_{S}}{2}\right)\right]\\ +A_{2}\left[2\cos^{4}\left(\frac{\varphi_{S}}{2}\right)\left(\cos^{2}\left(\frac{\varphi_{S}}{2}\right)+2\sin^{2}\left(\frac{\varphi_{S}}{2}\right)\right)+2\sin^{4}\left(\frac{\varphi_{S}}{2}\right)\left(\sin^{2}\left(\frac{\varphi_{S}}{2}\right)+2\cos^{2}\left(\frac{\varphi_{S}}{2}\right)\right)\right] \end{array}$$

where:

$$\begin{array}{c} A_{1}=N_{1}N_{2}{\left(1-x\right)}^{2}x^{2}\eta_{I}^{2}\eta_{s}^{2}\\ A_{2}=4N_{1}N_{2}{\left(1-x\right)}^{2}x^{3}\left[1-{\left(1-\eta_{I}\right)}^{2}\right]\left[1-{\left(1-\eta_{s}\right)}^{2}\right] \end{array}$$

In the previous relationship, we have set $x_{1}=x_{2}=x$; and, $L\left(1_{I1}\right)=L\left(1_{I2}\right)=\eta_{I}$, $L\left(2_{I1}\right)=L\left(2_{I2}\right)=1-{\left(1-\eta_{I}\right)}^{2}$, $L\left(1_{s1}\right)=L\left(1_{s2}\right)=\eta_{s}$, $L\left(2_{s1}\right)=L\left(2_{s2}\right)=1-{\left(1-\eta_{s}\right)}^{2}$.

The next step is to increase the gyro resolution further by measuring higher order coincidences. We suggest employment of six single-photon detectors SPAD-i (i = 1,2,3,4,5,6), as depicted in Figure 1c,d. We examine the coincidence of clicking six detectors i.e., the probability given by:

(14) $$P_{6}^{Ind\left(Dis\right)}={\langle 1|}_{D2}{\langle 1|}_{D3}{\langle 1|}_{D4}{\langle 1|}_{D5}\left({\hat{\rho }}^{Ind\left(Dis\right)}\right){|1\rangle }_{D2}{|1\rangle }_{D3}{|1\rangle }_{D4}{|1\rangle }_{D5}$$

With reference to the Figure 1c,d, we indicate with $v_{1}$ and $v_{2}$ the vacuum ports at the beam splitters BS-5 and BS-6

Therefore, from Equations (10) and (14) and for architecture of Figure 1c, we obtain:

(15) $$P_{6}^{\left(Ind\right)}=A_{3}r^{4}t^{4}\cdot {\left|\begin{array}{c} {\left[\sin^{2}\left(\frac{\varphi_{S}}{2}\right)+\cos^{2}\left(\frac{\varphi_{S}}{2}\right)\cos^{2}\left(2\theta_{BS}\right)\right]}^{2}+\cos^{4}\left(\frac{\varphi_{S}}{2}\right)\sin^{4}\left(2\theta_{BS}\right)-\\ 4\cos^{2}\left(\frac{\varphi_{S}}{2}\right)\sin^{2}\left(2\theta_{BS}\right)\cdot \left[\sin^{2}\left(\frac{\varphi_{S}}{2}\right)+\cos^{2}\left(\frac{\varphi_{S}}{2}\right)\cos^{2}\left(2\theta_{BS}\right)\right] \end{array}\right|}^{2}$$

(16) $$P_{6}^{Dis}=A_{3}r^{4}t^{4}[\begin{array}{c} {\left[\sin^{2}\left(\frac{\varphi_{S}}{2}\right)+\cos^{2}\left(\frac{\varphi_{S}}{2}\right)\cos^{2}\left(2\theta_{BS}\right)\right]}^{4}+\cos^{8}\left(\frac{\varphi_{S}}{2}\right)\sin^{8}\left(2\theta_{BS}\right)+\\ 4\cos^{4}\left(\frac{\varphi_{S}}{2}\right)\sin^{4}\left(2\theta_{BS}\right)\cdot {\left[\sin^{2}\left(\frac{\varphi_{S}}{2}\right)+\cos^{2}\left(\frac{\varphi_{S}}{2}\right)\cos^{2}\left(2\theta_{BS}\right)\right]}^{2} \end{array}]$$

where

$$A_{3}=4N_{1}N_{2}{\left(1-x\right)}^{2}x^{4}\eta_{s}^{4}{\left[1-{\left(1-\eta_{I}\right)}^{2}\right]}^{2}$$

and r and t represent the Cross and Bar amplitude coefficient of the beam splitters BS-5 and BS-6, respectively.

Moreover, Equations (15) and (16) take the effect of the splitting ratio ($\theta_{BS}$) of the beam splitters BS-3 and BS-4 into account. Similarly, for the architecture of Figure 1d, we obtain Equations (17) and (18).

Under the indistinguishable condition and assuming the optimized case 50:50 for the splitting ratio of BS-3 and BS-4, Equations (15) and (17) show that the fringe patterns are proportional to ${\left[1+3\cos\left(2\varphi_{S}\right)\right]}^{2}$ and ${\left[\sin\left(2\varphi_{S}\right)\right]}^{2}$ for Figure 1c,d, respectively. Thus, a reduction of the fringe period is obtained, which results in an increase of the resolution of four times with respect to the classical gyro performance at room temperature, although requiring higher power than the first two architectures, in order to obtain multi pair generation. As a result, the nonlinear effects (SPM) affect the phase matching condition Δβ = 0. However, the exact frequency shift, Δf, to achieve the generalized phase matching condition, $\kappa =\Delta \beta +2\gamma P_{0}=0$, can be always found, by setting the parameter Δβ to an opportune negative value, depending on the pump power. Our simulations record that, when changing $P_{0}$ from 10 mW to 300 mW, the condition κ=0 can be found, by ranging Δf between 0.2 and 1.148 THz. Moreover, we believe that the very small temperature change does not induce any significant detrimental effect on the operative phase matching condition. We have estimated that a temperature change ΔT = 1 K, induces Δf changes from 0.2 and 3.4 THz. However, this effect can be mitigated using a thermal controller.

**Figure 6:**
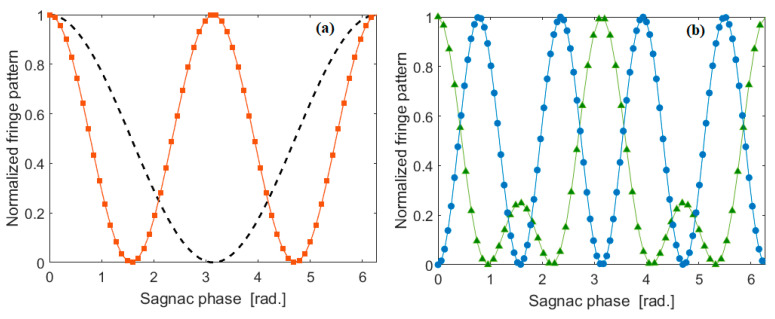
Normalized fringe pattern as a function of the Sagnac phase, for the integrated gyro architectures. (**a**) Dashed line: Classic interference; red square: two photon coincidence, Equation (8) or four-fold coincidence for indistinguishable photons, Equation (12); (**b**) Green triangles: six coincidence for indistinguishable photons, Equation (15); Blue circles: six coincidence for indistinguishable photons, Equation (17).

The theory before described is summarized in Figure 6 where the normalized fringe pattern as a function of the Sagnac phase is shown for all cases previously discussed. The plot records that for indistinguishable photons the visibility is theoretically 100%.

(17) $$P_{6}^{\left(Ind\right)}=9A_{3}{\left(r^{3}t^{2}\right)}^{2}{\left|B_{1}+B_{2}\right|}^{2}$$

where

$$B_{1}=\left[\cos^{2}\left(\frac{\varphi_{S}}{2}\right)\cos^{2}\left(2\theta_{BS}\right)+\sin^{2}\left(\frac{\varphi_{S}}{2}\right)\right]\cdot \left[-j\sin\left(\frac{\varphi_{S}}{2}\right)+\cos\left(\frac{\varphi_{S}}{2}\right)\cos\left(2\theta_{BS}\right)\right]\cdot \cos\left(\frac{\varphi_{S}}{2}\right)\sin\left(2\theta_{BS}\right)$$

$$\;B_{2}=-\left[-j\sin\left(\frac{\varphi_{S}}{2}\right)+\cos\left(\frac{\varphi_{S}}{2}\right)\cos\left(2\theta_{BS}\right)\right]\cdot \cos^{3}\left(\frac{\varphi_{S}}{2}\right)\sin^{3}\left(2\theta_{BS}\right)\;$$

(18) $$P_{6}^{Dis}=3A_{3}{\left(r^{3}t^{2}\right)}^{2}\left[B_{3}+B_{4}\right]$$

where

$$B_{3}={\left[\cos^{2}\left(\frac{\varphi_{S}}{2}\right)\cos^{2}\left(2\theta_{BS}\right)+\sin^{2}\left(\frac{\varphi_{S}}{2}\right)\right]}^{2}\cdot {\left[-j\sin\left(\frac{\varphi_{S}}{2}\right)+\cos\left(\frac{\varphi_{S}}{2}\right)\cos\left(2\theta_{BS}\right)\right]}^{2}\cdot \cos^{2}\left(\frac{\varphi_{S}}{2}\right)\sin^{2}\left(2\theta_{BS}\right)$$

$$B_{4}={\left[-j\sin\left(\frac{\varphi_{S}}{2}\right)+\cos\left(\frac{\varphi_{S}}{2}\right)\cos\left(2\theta_{BS}\right)\right]}^{2}\cdot \cos^{6}\left(\frac{\varphi_{S}}{2}\right)\sin^{6}\left(2\theta_{BS}\right)$$

**Figure 7:**
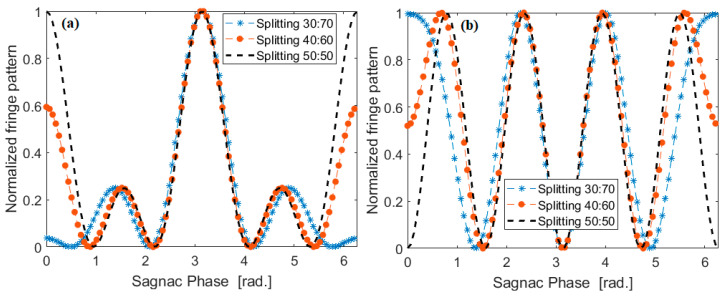
Normalized fringe pattern versus the Sagnac phase, for different values of the splitting ratio of BS-3 and BS-4; (**a**) architecture of Figure 1b; (**b**) architecture of Figure 1c.

We see that the architecture of Figure 1c shows a reduction in the period fringes, but it develops smaller peaks. However, this aspect is solved by the architecture of Figure 1d, where the pure four-fold increasing of the resolution is obtained having all peaks at the same height. Finally, the detrimental effect of the splitting ratio being different from 50:50 is shown in Figure 7, where the normalized fringe pattern versus the Sagnac phase, when assuming a splitting ratio equal to 30:70, 40:60, and 50:50 (optimum), is plotted for the devices of Figure 1c,d, respectively. For the coincidence measurements, all of the SPADs must be connected to a picosecond-resolution time tagger, which records the photons arrival times. The post-processing of these times-of-arrival is applied to identify the fringe pattern. At this stage, it is difficult to estimate the acquisition time without any experimental feedback, being strongly dependent on environmental conditions and the measured efficiency of the photodetectors. However, from an analysis of the literature [50] the acquisition time could range from 10 s to 50 s. In this context, it represents the main drawback with respect to the classical approach. Indeed, the review papers indicate that the integration time for classical IFOG is around 1 s. We believe that a three-dimensional (3D) rotation sensor can be made easily using three of these gyro chips by orienting the chips at 90 degrees to each other, that is the plane of the chip is XY for chip 1, YZ for chip 2, and ZX for chip 3.

## 5. Conclusions

For operation at 1550 nm, chip-scale room-temperature integrated quantum interferometric gyro architectures have been proposed for foundry implementation in the Group IV SOI/GeSn technological platform. The focus of this work has been to demonstrate that integrated architectures can induce super-resolution with respect to what is obtained with the classic approach. Basically, the proposed devices present the one-chip co-integration of entangled photon sources (i.e., spiraled waveguides or micro-ring resonators in which signal-idler pairs are generated by means of the SFWM process), beam splitters, MZI, phase shifter, gyro interferometer, and an array of single-photon detectors (SPADs). Moreover, the presented architectures can operate either with degenerate and non-degenerate SFWM effect. In the first case, the split or bunched (N00N) state has been used to measure the two-fold coincidences, recording an improvement of two times in the gyro resolution. Alternatively, two different gyro configurations that are based on two-mode squeezing (induced by non-degenerate SFWM) have been proposed to operate with four-fold and higher order coincidences. The theoretical investigations have demonstrated that an increase of a factor of four is also possible with a visibility of 100% for indistinguishable photons. We think that experiments should be feasible because many two-photon and four-photon interference effects have been observed in quantum Silicon photonics.

## Figures and Tables

**Table 1:** Design Parameters for SOI and SiN platforms.

Parameters	Si/SiO_2_ W = 500 nm; H = 220 nm	Si/SiO_2_ W = 500 nm; H = 250 nm	Si_3_N_4_/SiO_2_ W = 1060 nm; H = 600 nm	Si-enriched SiN/SiO_2_ W = 790 nm; H = 600 nm
$\;\lambda_{0}$ [nm]	1550	1550	1551	1552
$\;\Delta f_{0,1}$ [GHz]@ $P_{0}$ = 10 mW	240	215	985	330
Photon pair generation rate [Photon/Hz·s]	2.94 × 10^−4^ @ L = 10 mm, $\Delta f=\Delta f_{0,1}\;$	2.53 × 10^−4^ @ L = 10 mm, $\Delta f=\Delta f_{0,1}\;$	1.83 × 10^−7^ @ L = 30 mm, $\Delta f=\Delta f_{0,1}\;$	2.03 × 10^−6^ @ L = 20 mm, $\Delta f=\Delta f_{0,1}\;$
FWHM [THz]	2.52	2.31	8.74	8.4

## Supplementary Materials

The following are available online at https://www.mdpi.com/1424-8220/20/12/3476/s1, Figure S1: Schematic top view of proposed quantum optical gyro architecture; Figure S2: Beam splitter coupling ratio as a function of the coupling region length (interaction length), for different values of gap between the two strip SOI waveguides; Figure S3: Squared norm of the electric field in the directional coupler. The dashed line shows the length where the 50% of coupling ratio is achieved; Figure S4: (a) Drop spectrum of the MRR pump filter; (b) Drop and Pass spectrum around $\lambda_{p}$(Zoom-in); Figure S5: (a) Drop spectrum of the MRR Idler filter; (b) Drop and Pass spectrum around $\lambda_{I}$(Zoom-in).
