# Supplementary material for: On-Chip Group-IV Heisenberg-Limited Sagnac Interferometric Gyroscope at Room Temperature
Source: Sensors (Basel). 2020 Jun 19;20(12):3476. doi: 10.3390/s20123476 (PMC7349385; doi:10.3390/s20123476)
Supplement: Supplementary file 1 [file sensors-20-03476-s001.pdf]

sensors

MDPI

Article

On-Chip Group-IV Heisenberg-Limited Sagnac Interferometric Gyroscope at Room Temperature

Francesco De Leonardis [1], Richard Soref [2], Martino De Carlo [1], and Vittorio M. N. Passaro [1,*]

[1] Dipartimento di Ingegneria Elettrica e dell'Informazione, Politecnico di Bari, Via Edoardo Orabona n. 4, 70125 Bari, Italy; francesco.deleonardis@poliba.it (F.D.L.); martino.decarlo@poliba.it (M.D.C.)

[2] Department of Engineering, University of Massachusetts Boston, Boston, MA 02125, USA; richard.soref@umb.edu (R.S.)

* Correspondence: vittorio.passaro@poliba.it (V.M.N.P.)

Received: 12 May 2020; Accepted: 17 June 2020; Published: 19 June 2020

Higher-order coincidence

$$\begin{bmatrix} c^\dagger \\ d^\dagger \end{bmatrix} = \begin{bmatrix} -j\sin\left(\frac{\varphi_S}{2}\right) + \\ +\cos\left(\frac{\varphi_S}{2}\right)\cos(2\theta_{BS}) & j\cos\left(\frac{\varphi_S}{2}\right)\sin(2\theta_{BS}) \\ j\cos\left(\frac{\varphi_S}{2}\right)\sin(2\theta_{BS}) & j\sin\left(\frac{\varphi_S}{2}\right) + \\ & +\cos\left(\frac{\varphi_S}{2}\right)\cos(2\theta_{BS}) \end{bmatrix} \begin{bmatrix} a^\dagger \\ b^\dagger \end{bmatrix} = \mathbf{U} \begin{bmatrix} a^\dagger \\ b^\dagger \end{bmatrix} = \begin{bmatrix} U_{11} & U_{12} \\ U_{21} & U_{22} \end{bmatrix} \begin{bmatrix} a^\dagger \\ b^\dagger \end{bmatrix} \tag{S1}$$

where the global phase $e^{j\varphi_S/2}$ has been dropped, and the phase θ_{BS} determines the splitting ratio of the beam splitter BS-3 and BS-4, assumed to be equal. Similarly, the relationship between the operators for BS-3 and BS-4 are given by:

$$\begin{bmatrix} v_1^\dagger \\ e^\dagger \end{bmatrix} = \begin{bmatrix} t & jr \\ jr & t \end{bmatrix} \begin{bmatrix} m^\dagger \\ g^\dagger \end{bmatrix} = \mathbf{M} \begin{bmatrix} m^\dagger \\ g^\dagger \end{bmatrix} = \begin{bmatrix} M_{11} & M_{12} \\ M_{21} & M_{22} \end{bmatrix} \begin{bmatrix} m^\dagger \\ g^\dagger \end{bmatrix} \tag{S2}$$

$$\begin{bmatrix} v_2^\dagger \\ m^\dagger \end{bmatrix} = \begin{bmatrix} t & jr \\ jr & t \end{bmatrix} \begin{bmatrix} e^\dagger \\ f^\dagger \end{bmatrix} = \mathbf{M} \begin{bmatrix} e^\dagger \\ f^\dagger \end{bmatrix} = \begin{bmatrix} M_{11} & M_{12} \\ M_{21} & M_{22} \end{bmatrix} \begin{bmatrix} e^\dagger \\ f^\dagger \end{bmatrix} \tag{S3}$$

where and r and t represent the Cross and Bar amplitude coefficients of the beam splitters BS-5 and BS-6, respectively. The terms v_1 and v_2 are operators at the vacuum ports of the beam splitters BS-5 and BS-6, respectively.

Substituting Equations. (S2) and (S3) in Equation. (S1), we obtain:

$$a^\dagger = U_{11}\left(M_{21}^2 e^\dagger + M_{21}M_{22} f^\dagger + M_{22} g^\dagger\right) + U_{12} d^\dagger \tag{S4}$$

$$b^\dagger = U_{21}\left(M_{21}^2 e^\dagger + M_{21}M_{22} f^\dagger + M_{22} g^\dagger\right) + U_{22} d^+ \tag{S5}$$

The meaning of all operators is sketched in Figure. S1. Moreover, Equations. (S4) and (S5) hold for the hypothesis of indistinguishable emitted photons, where the initial state at the input of the gyro interferometer can be written as $|\psi_{in}\rangle a^\dagger b^\dagger |00\rangle$. Now, if we assume that the photons are generated in separate temporal modes (distinguishable), then the input state must be written as $|\psi_{in}\rangle a^\dagger b'^\dagger |00\rangle$,

where $b'^{\dagger}$ indicates that the temporal mode is not overlapped to $a^{\dagger}$. In this context, Equation. (S5) must be re-written by placing the prime symbol on all operators.

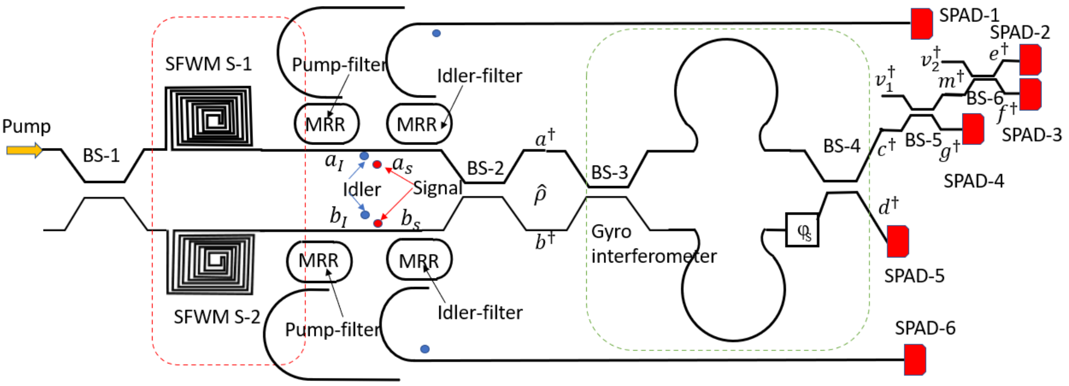

Figure S1. Schematic top view of proposed quantum optical gyro architecture.

We assume the idler photons generate from the sources S-1 and S-2 to be two heralding photons. As a result, the idler photons, detected at SPAD-1 and SPAD-6, are used to herald the presence of signal photons at SPAD-2, SPAD-3, SPAD-4 and SPAD-5. In this context, the reduced density matrix for the heralded signal photons for each source is:

$$\hat{\rho}_i = N_i\left(1-x_i\right)\left(x_i L\left(1_{Ii}\right)|1\rangle\langle 1|_{si} + x_i^2 L\left(2_{Ii}\right)|2\rangle\langle 2|_{si}\right) \quad \text{(S6)}$$

with $i = 1{,}2$. The term N_i is the normalization coefficient, x_i is the squeezing parameter, and $L(n_{Ii})$ takes into account the losses experienced by n idler photons emitted by the i-th SFWM source as well as the SPAD loss. In this context, the density matrix for the system with S1 and S2 is given by:

$$\hat{\rho} = \hat{\rho}_1 \otimes \hat{\rho}_2 = N_1 N_2\left(1-x_1\right)\left(1-x_2\right)\begin{pmatrix} x_1 L\left(1_{I1}\right)|1\rangle\langle 1|_{s1} + \\ x_1^2 L\left(2_{I1}\right)|2\rangle\langle 2|_{s1} \end{pmatrix} \otimes \begin{pmatrix} x_2 L\left(1_{I2}\right)|1\rangle\langle 1|_{s2} + \\ x_2^2 L\left(2_{I2}\right)|2\rangle\langle 2|_{s2} \end{pmatrix} \quad \text{(S7)}$$

where $I_1(s_1)$ and $I_2(s_2)$ indicate the idler(signal) photon generated from the source S-1 and S-2, respectively. Thus, with reference to the Figure. S1, the probability of detecting the coincidence of clicking six detectors is:

$$P_6^{Ind(Dis)} = \langle 1|_{D2}\langle 1|_{D3}\langle 1|_{D4}\langle 1|_{D5}\left(\hat{\rho}^{Ind(Dis)}\right)|1\rangle_{D2}|1\rangle_{D3}|1\rangle_{D4}|1\rangle_{D5} \quad \text{(S8)}$$

where the superscripts Ind and Dis mean the indistinguishable and distinguishable emitted photons. Hereafter we particularize the calculations for the indistinguishable case. It is worth outlining that if the sources emit at most two photon pairs, then only one term of (S7) (indicated in the following with $\hat{\rho}_6$) will contribute to the six detectors clicking. Therefore, we have:

$$\hat{\rho}_6 = A_3\left(|2\rangle\langle 2|_{s1} \otimes |2\rangle\langle 2|_{s2}\right) \quad \text{(S9)}$$

where

$$A_3 = N_1 N_2\left(1-x_1\right)\left(1-x_2\right)x_1^2 x_2^2 P\left(2_{I1}\right)P\left(2_{I2}\right) \quad \text{(S10)}$$

We set:

$$T_1 = U_{11}M_{21} = jr\left[-j\sin\left(\frac{\varphi_S}{2}\right)+\cos\left(\frac{\varphi_S}{2}\right)\cos(2\theta_{BS})\right];\ T_2 = U_{11}M_{22} = t\left[-j\sin\left(\frac{\varphi_S}{2}\right)+\cos\left(\frac{\varphi_S}{2}\right)\cos(2\theta_{BS})\right]$$
$$T_3 = U_{12}M_{21} = -r\cos\left(\frac{\varphi_S}{2}\right)\sin(2\theta_{BS});\ T_4 = U_{12}M_{22} = jt\cos\left(\frac{\varphi_S}{2}\right)\sin(2\theta_{BS});\ T_5 = U_{21}M_{21} = -r\cos\left(\frac{\varphi_S}{2}\right)\sin(2\theta_{BS}) \quad \text{(S11)}$$
$$T_6 = U_{21}M_{22} = jt\cos\left(\frac{\varphi_S}{2}\right)\sin(2\theta_{BS});\ T_7 = U_{22}M_{21} = jr\left[j\sin\left(\frac{\varphi_S}{2}\right)+\cos\left(\frac{\varphi_S}{2}\right)\cos(2\theta_{BS})\right];$$
$$T_8 = U_{22}M_{22} = t\left[j\sin\left(\frac{\varphi_S}{2}\right)+\cos\left(\frac{\varphi_S}{2}\right)\cos(2\theta_{BS})\right]$$

Then,

$$\hat{\rho}_6^{Ind} =$$
$$\frac{1}{4}A_3\left\{\begin{array}{l}\left[(T_1e^\dagger)^2+(T_2f^\dagger)^2+(T_3g^\dagger)^2+(T_4l^\dagger)^2+2T_1T_2e^\dagger f^\dagger+2T_1T_3e^\dagger g^\dagger+2T_2T_3f^\dagger g^\dagger+2T_1T_4e^\dagger l^\dagger+2T_2T_4f^\dagger l^\dagger+2T_3T_4g^\dagger l^\dagger\right]\cdot\\ \left[(T_5e^\dagger)^2+(T_6f^\dagger)^2+(T_7g^\dagger)^2+(T_8l^\dagger)^2+2T_5T_6e^\dagger f^\dagger+2T_5T_7e^\dagger g^\dagger+2T_6T_7f^\dagger g^\dagger+2T_5T_8e^\dagger l^\dagger+2T_6T_8f^\dagger l^\dagger+2T_7T_8g^\dagger l^\dagger\right]\\ |0\rangle\langle 0|_{D2}\otimes|0\rangle\langle 0|_{D3}\otimes|0\rangle\langle 0|_{D4}\otimes|0\rangle\langle 0|_{D5}\\ \left[(T_1^*e)^2+(T_2^*f)^2+(T_3^*g)^2+(T_4^*l)^2+2T_1^*T_2^*ef+2T_1^*T_3^*eg+2T_2^*T_3^*fg+2T_1^*T_4^*el+2T_2^*T_4^*fl+2T_3^*T_4^*gl\right]\cdot\\ \left[(T_5^*e)^2+(T_6^*f)^2+(T_7^*g)^2+(T_8^*l)^2+2T_5^*T_6^*ef+2T_5^*T_7^*eg+2T_6^*T_7^*fg+2T_5^*T_8^*el+2T_6^*T_8^*fl+2T_7^*T_8^*gl\right]\end{array}\right\} \quad \text{(S12)}$$

Substituting Equation. (S12) into Equation. (S8) we obtain:

$$P_{6F} = 4A_3L(1_{D2})L(1_{D3})L(1_{D4})L(1_{D5})\left|T_1T_2T_7T_8+T_1T_3T_6T_8+T_2T_3T_5T_8+T_1T_4T_6T_7+T_2T_4T_5T_7+T_3T_4T_5T_6\right|^2 =$$
$$4A_3L(1_{D2})L(1_{D3})L(1_{D4})L(1_{D5})9\left(r^3t^2\right)^2\left|\begin{array}{l}\left[\cos^2\left(\frac{\varphi_S}{2}\right)\cos^2(2\theta_{BS})+\sin^2\left(\frac{\varphi_S}{2}\right)\right]\left[-j\sin\left(\frac{\varphi_S}{2}\right)+\cos\left(\frac{\varphi_S}{2}\right)\cos(2\theta_{BS})\right]\cdot\\ \cdot\cos\left(\frac{\varphi_S}{2}\right)\sin(2\theta_{BS})\\ -\left[-j\sin\left(\frac{\varphi_S}{2}\right)+\cos\left(\frac{\varphi_S}{2}\right)\cos(2\theta_{BS})\right]\cos^3\left(\frac{\varphi_S}{2}\right)\sin^3(2\theta_{BS})\end{array}\right|^2 \quad \text{(S13)}$$

For the architecture of Figure. 1 (c) (main text), we have similar mathematical developments.

Photonic Splitters and Filters

In the gyro architectures proposed in the main text, we have designed the beam splitter by means of the 3D propagator software. The numerical results of the 3D eigenmode solver are summarized in Fig. S2, where, the splitting ratio is plotted as a function of the interaction length for different values of the waveguide gap, ranging between 80 nm and 120 nm. The curves record that a beam splitter with a coupling ratio of 50% can be obtained by fixing the interaction length equal to L_0= 23.5 μm and g_0=100 nm. Ranging the coupler length from 22.31 μm to 24.69 μm (with the gap fixed at g_0) the coupling ratio varies between 61.71% and 38.51 %. Ranging the gap between 90 nm and 110 nm (with the coupler length fixed at L_0) the coupling ratio varies between 22.68 % and 73.72 %.

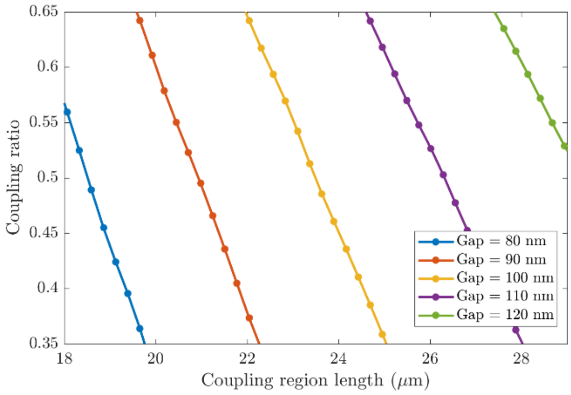

Figure S2. Beam splitter coupling ratio as a function of the coupling region length (interaction length), for different values of gap between the two strip SOI waveguides.

In this context, Figure. S3 shows the squared norm of the electric field in the directional coupler. The dashed line shows the length where the 50% coupling ratio is achieved (L_0 = 23.5 µm).

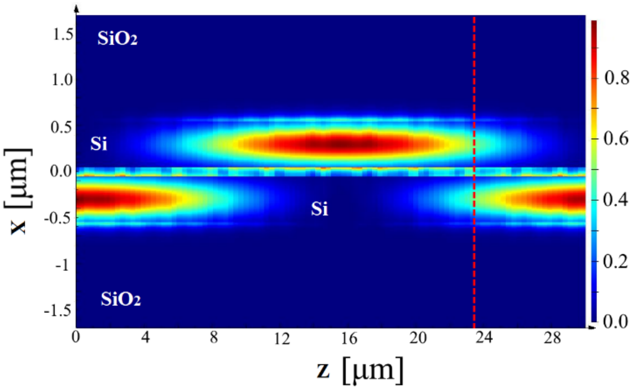

Figure S3. Squared norm of the electric field in the directional coupler. The dashed line shows the length where the 50% of coupling ratio is achieved.

In order to realize the full integration of the gyro chip, we think that a micro-ring racetrack resonator (MRR) coupled on both sides to silicon bus waveguides, can be considered as an efficient choice, in order to filter out the residual pump. Moreover, the architectures of Figures. 1 (b)-(d) (main text) include two micro-ring resonators in order to filter out the idler photons generate from the sources S-1 and S-2, in order to herald the signal photons. In this context, a possible design rule for both filters can be:

$$FSR_{pump-filter} > \left|\Delta f_{0,1}\right| \quad \text{(S14)}$$

$$FSR_{Idler-filter} > 2\left|\Delta f_{0,1}\right| \quad \text{(S15)}$$

where $FSR = \bar{\lambda}/(n_g L_{MRR})$. The terms n_g and L_{MRR} are the group refractive index and the micro-ring racetrack circumference, respectively. The wavelength $\bar{\lambda}$=λ_p=1550 nm, or $\bar{\lambda}$=λ_I=1548.1 nm.

The index distribution and the Sellmeier equation for Si and SiO_2 are fed in the FEM software to determine the effective refractive index and the group refractive index as a function of the wavelength. These functions are then inputs to the transfer matrix of the micro-ring resonator in order to theoretically predict the spectral response. The simulations record n_g= 4.08 around 1550 nm. Moreover, according to Equations. (S14) and (S15), $L_{MRR}(pump-filter)$ <306.5 µm and $L_{MRR}(Idler-filter)$<127.31 µm. Figures S4 and S5 show the spectrum for the pump and Idler filters, respectively. In the simulations we have assumed the coupling factor between the racetrack and the bus waveguides equal to 1% and$L_{MRR}(pump-filter)$=188.5 µm and $L_{MRR}(Idler-filter)$<62.32

µm, respectively. The simulations record a FWHM of about 0.012 and 0.026 nm for the pump and Idler filters, respectively.

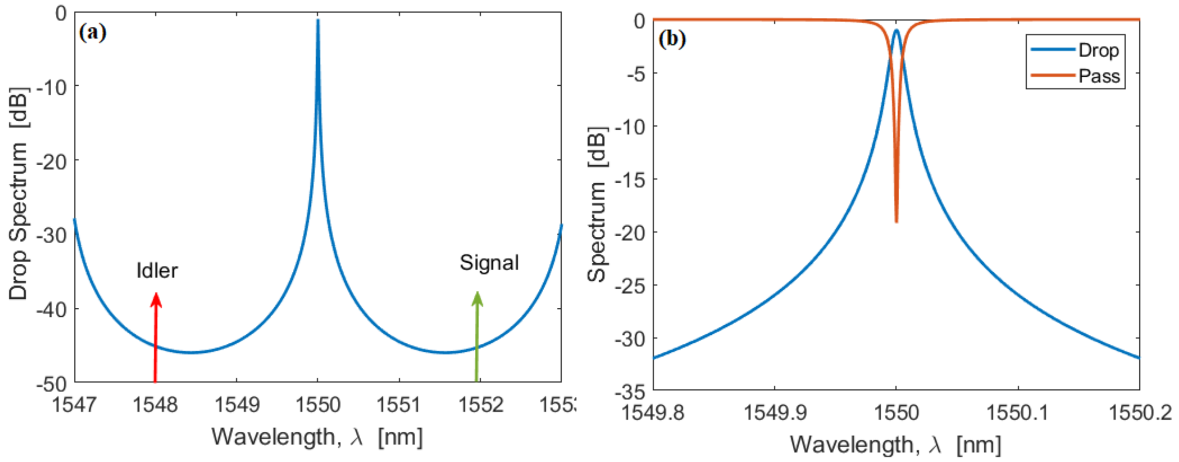

Figure S4. Drop spectrum of the MRR pump filter; (**b**) Drop and Pass spectrum around λ_p(Zoom-in).

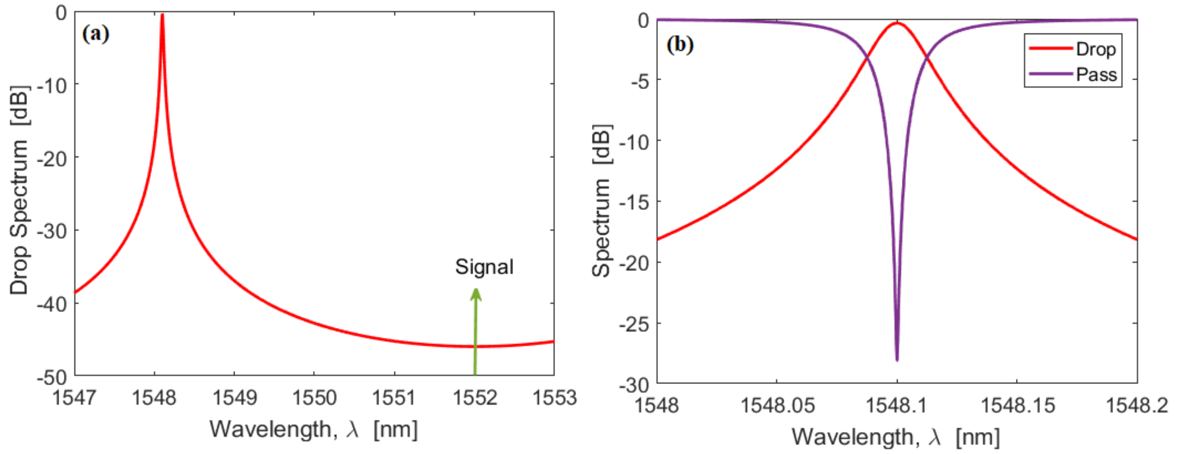

Figure S5. (**a**) Drop spectrum of the MRR Idler filter; (**b**) Drop and Pass spectrum around λ_I(Zoom-in).
